# Porcine rotavirus B as primary causative agent of diarrhea outbreaks in newborn piglets

**DOI:** 10.1038/s41598-020-78797-y

**Published:** 2020-12-15

**Authors:** Flavia Megumi Miyabe, Alais Maria Dall Agnol, Raquel Arruda Leme, Thalita Evani Silva Oliveira, Selwyn Arlington Headley, Thiago Fernandes, Admilton Gonçalves de Oliveira, Alice Fernandes Alfieri, Amauri Alcindo Alfieri

**Affiliations:** 1 Laboratory of Animal Virology, Department of Preventive Veterinary Medicine, Universidade Estadual de Londrina, PO Box 10011, Londrina, Paraná 86057-970 Brazil; 2 Multi-User Animal Health Laboratory-Molecular Biology Unit, Department of Preventive Veterinary Medicine, Universidade Estadual de Londrina, Londrina, Paraná Brazil; 3 Laboratory of Animal Pathology, Department of Veterinary Preventive Medicine, Universidade Estadual de Londrina, Londrina, Paraná Brazil; 4 Laboratory of Electron Microscopy, Department of Microbiology, Universidade Estadual de Londrina, Londrina, Paraná Brazil

**Keywords:** Microbiology, Virology, Rotavirus, Molecular biology

## Abstract

Rotavirus (RV) is considered a major cause of acute viral gastroenteritis in young animals. RV is classified into nine species, five of which have been identified in pigs. Most studies worldwide have highlighted diarrhoea outbreaks caused by RVA, which is considered the most important RV species. In the present study, we described the detection and characterization of porcine RVB as a primary causative agent of diarrhoea outbreaks in pig herds in Brazil. The study showed a high frequency (64/90; 71.1%) of RVB diagnosis in newborn piglets associated with marked histopathological lesions in the small intestines. Phylogenetic analysis of the VP7 gene of wild-type RVB strains revealed a high diversity of G genotypes circulating in one geographic region of Brazil. Our findings suggest that RVB may be considered an important primary enteric pathogen in piglets and should be included in the routine differential diagnosis of enteric diseases in piglets.

## Introduction

Rotaviruses (RV) are members of the *Reoviridae* family and a major cause of acute viral gastroenteritis in young animals, including piglets^1^. RV is a non-enveloped virus and its genome is composed of 11 segments of double-stranded RNA^2^. RV is classified into nine species (A–I), on the basis of the antigenic and genetic characteristics of viral protein 6 (VP6)^3,4^. A new tentative species J has been recently identified in bats from Serbia^5^. Currently, five of the nine species of RV have been identified in pigs (RVA, RVB, RVC, RVE, and RVH)^6^.

RVA was the first RV species to be identified and is considered the most important RV species because of its high prevalence and pathogenicity in both humans and animals^1,7^. In 2008, a nucleotide sequence-based classification system for the complete genome of RVA strains was developed; this system assigned specific genotypes to each of the 11 RV genome segments according to nucleotide (nt) and amino acid (aa) percent cutoff values^8,9^. The Rotavirus Classification Working Group (RCWG) maintains this classification and uses the abbreviations Gx-P[x]-Ix-Rx-Cx-Mx-Ax-Nx-Tx-Ex-Hx for the RV genes VP7-VP4-VP6-VP1-VP2-VP3-NSP1-NSP2-NSP3-NSP4-NSP5/6, respectively^8,10^.

Unlike RVA strains, which have been well characterized worldwide, RVB strains remain poorly characterized. In 2012, Marthaler et al.^11^ proposed a classification for G genotypes of RVB strains from the United States (USA), establishing 20 G genotypes; however, the complete genome of porcine RVB was described only recently^12^. Shepherd et al.^12^ established a provisional genome-based classification for RVB, suggesting and updating nt cutoff values for all RVB gene segments. According to this newly proposed classification, 26G, 5P, 13I, 5R, 5C, 5M, 8A, 10N, 6T, 4E, and 7H genotypes (VP7, VP4, VP6, VP1-VP3, NSP1-NSP5 genes, respectively) of RVB have been described, using nt cutoff values of 80%, 80%, 81%, 78%, 79%, 77%, 76%, 83%, 78%, 76%, and 79%, respectively.

Studies conducted in the USA and Brazil have shown high frequencies of RVB in piglets with diarrhoea, mostly in association with other RV species, such as RVA and RVC^6,7,11^. Thus, the role of RVB as a primary pathogen remains unclear, and until recently, this species was not considered a major causative agent of diarrhoea in piglets.

In the present study, we describe the detection of porcine RVB acting as a potential primary causative agent of neonatal diarrhoea outbreaks in several pig farms from the Central-West region of Brazil and the characterization of G genotypes of these Brazilian RVB field strains.

## Results

### PAGE and conventional RT-PCR

Forty-two of the 90 (46.7%) biological samples evaluated in this study displayed typical electropherotype of RVB species on polyacrylamide gel electrophoresis assay (PAGE), while 14 samples had inconclusive results. In the conventional RT-PCR assay, 64 of the 90 (71.1%) samples were positive for RVB; these corresponded to 46.2% (6/13) of the intestinal tissues and 75.3% (58/77) of the diarrheic faeces samples evaluated. Coinfections of RVB with RVA and RVH were identified, by conventional RT-PCR, in 2.22% (2/90) and 4.44% (4/90) of samples, respectively. All the following enteric viruses were negative in all samples by conventional RT-PCR: RVC, swine delta coronavirus (SDCoV), porcine epidemic diarrhoea virus (PEDV), transmissible gastroenteritis virus (TGEV), Seneca Valley virus (SVV), and porcine enterovirus (PEV).

### Histopathology

**Figure 1:**
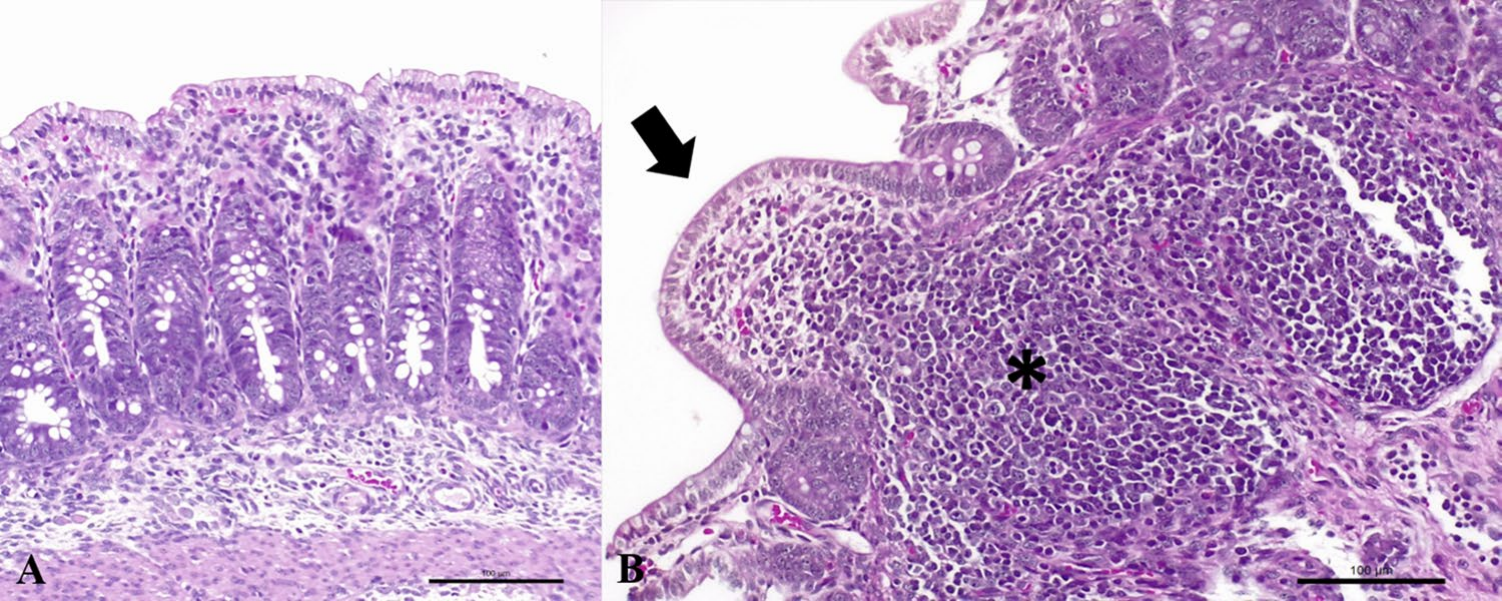
Histopathological findings in jejunum observed in newborn piglets naturally infected by Rotavirus B. **(A)** Observed marked atrophic enteritis with villous atrophy and blunting. **(B)** Severe atrophic enteritis with villous fusion (arrow) and hyperplasia of Peyer patches (asterisk). Hematoxylin & Eosin stain. Bar, **(A,B)** 100 μm. Magnification, × 40.

The histopathological findings from the jejunum revealed atrophic enteritis with marked villous atrophy and blunting, as well as reduced crypt depth, submucous lymphangiectasis, and hyperplasia of Peyer patches (Fig. 1).

### Transmission electron microscopy

The electron microscopy (EM) examination of a faecal sample revealed the presence of wheel-shaped viruses of approximately 70 nm, similar to RV-like particles (Supplementary Fig. S1).

### Virus isolation

Attempts at virus isolation from cell culture were performed using the same faecal sample that was analysed by EM; however, after nine blind passages in monolayer of MA-104 (African green monkey kidney) cells, no cytopathic effect (CPE) was visualized. Moreover, aliquots of all passages tested negative for RVB and other RV species (RVA, RVC and RVH), as well as SDCoV, PEDV, TGEV, SVV, and PEV in the RT-PCR assay.

### Real-time reverse transcription PCR assay (qRT-PCR)

The qRT-PCR assay developed in this study for quantification of RVB RNA in piglet faecal samples had a limit of detection (LOD) of 13.4 genomic copies/µL. The viral loads in diarrheic faecal samples varied from 7.6 × 10^2^ to 8.5 × 10^8^ genomic copies/g of faeces. The mean viral loads of the RVB-positive samples are described in terms of pig herd and sampling in Table 1. In pig herds A and B, the RVB viral load shed in the faeces of diarrheic piglets seems to have a cyclic pattern, with high and low viral loads observed in the four samplings during the outbreak.

**Table 1:** Distribution of total biological samples positives for RVB and respective viral load and G genotypes identified by pig herds and sampling.

Pig herd	Sampling	Month/year of sampling	RVB positive/total samples	Mean viral load (genomic copies/g of faeces)	G genotype
A	1	Aug/2017	¾	1.7 × 10^8^	G16
	2	Oct/2017	1/1	1.3 × 10^5^	–
	3	Feb/2018	5/5	1.7 × 10^8^	G16
	4	Aug/2018	0/5	Not tested	–
B	1	Aug/2017	5/6	2.0 × 10^8^	G16
	2	Oct/2017	1/1	2.5 × 10^5^	–
	3	Feb/2018	8/9	1.9 × 10^7^	G16
	4	Aug/2018	3/5	2.3 × 10^3^	–
C	1	Aug/2017	5/5	8.9 × 10^7^	G16
	2	Sep/2017	5/5	6.6 × 10^7^	G16
	3	Oct/2017	1/1	2.2 × 10^7^	G16
	4	Jun/2018	3/4	2.6 × 10^3^	G12
	5	Aug/2018	0/2	Not tested	–
D	1	Aug/2017	1/5	3.6 × 10^7^	G14
	2	Sep/2017	2/2	7.6 × 10^6^	–
	3	Oct/2017	1/3	4.3 × 10^5^	–
	4	Jun/2018	3/3	5.0 × 10^7^	G16
	5	Aug/2018	0/5	Not tested	–
E	1	Sep/2017	10/12	1.1 × 10^7^	G12
F	1	Oct/2017	1/1	3.5 × 10^7^	G20
G	1	Jun/2018	1/1	9.1 × 10^7^	G16
H	1	Jun/2018	5/5	2.1 × 10^3^	G12
Total			64/90		

### Sequencing and phylogenetic analysis

Comparative sequence analysis of the VP7 gene of 16 RVB field strains found in this study and the 26 known G genotypes of RVB^12^ revealed that at least four different G genotypes (G12, G14, G16, and G20) were circulating in these outbreaks.

The GO-949, GO-951, GO-1099, and GO-1113 strains displayed higher (77.1 to 80.4%) nt identity with other G12 strains; the GO-936 strain displayed 80 to 84.4% nt identity with the G14 strains; and the GO-992 strain displayed 81.3 to 85.4% nt identity with the G20 strains. Last, the GO-908, GO-923, GO-968, GO-970, GO-983, GO-1045, GO-1053, GO-1119, GO-1123, and GO-1127 strains showed higher (81.5 to 83.9%) nt identity with the G16 strains, which was the most prevalent G genotype found in this study (Supplementary Table S2 shows the identity matrix of RVB strains described in this study and representative strains of the 26 previously identified RVB G genotypes^12^).

**Figure 2:**
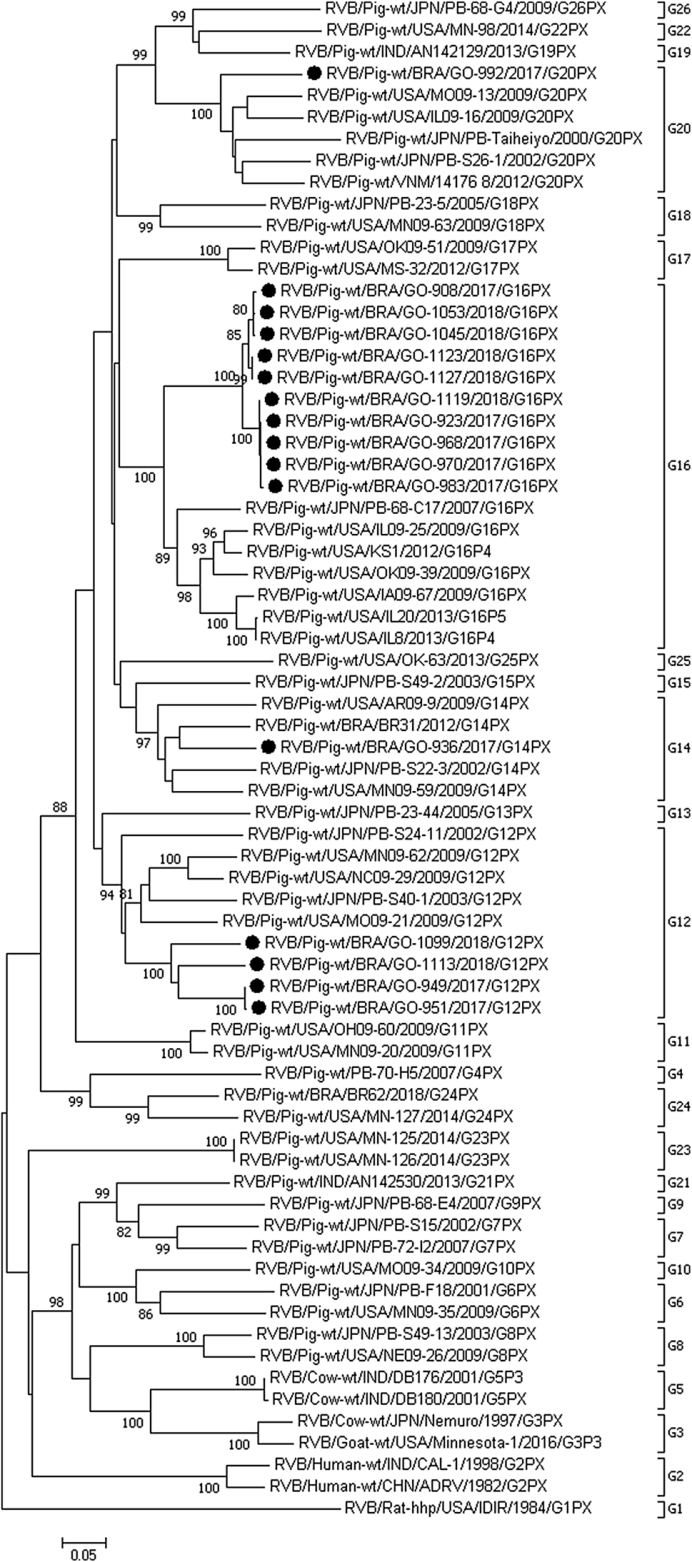
Phylogenetic tree based on nucleotide sequences (700 nt) of the VP7 gene of porcine RVB strains described in this study and representative strains of the 26 previously identified RVB G genotypes^12^. The tree was constructed using the neighbor-joining method based on the Kimura 2-parameter model. The scale bar indicates nt substitutions per site. The bootstrap values (1000 replicates) are shown at the branch nodes (values < 80% not shown). The Brazilian porcine RVB strains are marked with filled circles.

In the phylogenetic tree (Fig. 2), the G16 RVB Brazilian field strains clustered in a different branch in comparison with other G16 strains; however, using the nt cutoff value of 80%, the sequence identity matrix revealed that the strains belong to the same G genotype. Comparison among the Brazilian G12 strains showed that the strains share 85.4–99.8% nt identity, while the strains belonging to the G16 genotype shared high (96.6 to 100%) nt identity.

As the outbreaks occurred over a period of one year, some samples collected from pig herds were from different peaks of diarrhoea episodes, and it was possible to longitudinally analyse these RVB strains. Two different G genotypes were found circulating in pig herds C and D over the sampling period. In pig herd C, the genotype G16 was detected in the first samplings, while in the last sampling, the G12 genotype was identified. Alternatively, in pig herd D, the genotype G14 was identified in the first samplings, while the genotype G16 was detected in the last samplings (Table 1).

## Discussion

Although RVA has been reported as the most prevalent RV species in both humans and animals, porcine RVB was previously described in several regions worldwide, but usually in sporadic cases^13–15^. Studies conducted in Japan, the USA, and Brazil have shown porcine RVB frequencies of 25.9%, 46.8%, and 32%, respectively^6,11,16^. However, in these previous reports, RVB was detected mostly in association with other RV species, such as RVA, RVC, and RVH. In the present study, we detected porcine RVB in more than 70% (64/90) of the diarrheic faeces/ intestine tissues, with the highest RVB frequency reported thus far. In addition, most (58/90; 64.4%) of the diarrheic faecal samples were positive only for RVB, and coinfections with other RV species were detected in only 6 (6.7%) faecal samples. Furthermore, other important enteric viruses (RVC, SDCoV, PEDV, TGEV, SVV, and PEV) were not detected in these samples, and diarrhoea outbreaks were unresponsive to treatment with wide-spectrum antibiotics.

The high mortality rate observed in the outbreaks reported in this study suggests the involvement of pathogenic RVB field strains. The marked villous atrophy observed in the small intestines of affected piglets resulted in acute malabsorption and diarrhoea, which may have led to the high mortality of piglets. Infections caused by SVV and TGEV may produce similar lesions in the intestines of affected pigs^17–19^. Nevertheless, in this study, these viral disease pathogens were not identified by RT-PCR. It must be highlighted that RV replicates predominantly in the cytoplasm of villous enterocytes of the jejunum and ileum and induces cell lysis, villous blunting and atrophy, as observed by histopathology. Severe lesions are mostly associated with the RVA and RVC species^20,21^; however, our results suggest that RVB may also induce marked lesions in the small intestine of affected piglets and can be considered an important enteric pathogen in pigs, acting as a primary aetiologic agent of neonatal diarrhoea outbreaks.

Kuga et al.^16^ and Marthaler et al.^11^ observed the highest frequencies of RVB in animals > 55 days of age or fattening pigs, implying that RVB detection in clinical samples appears to increase with age. On the other hand, Alekseev et al.^22^ detected RVB causing a diarrhoea outbreak in suckling piglets with 3 to 5 days of age in Russia, corroborating our results. The diarrhoea outbreaks described in our study affected only piglets < 20 days of age, mostly newborn piglets, suggesting that RVB can be highly detected in all age groups.

Some authors have suggested that the immune pressure induced by mass vaccination against RVA may have driven the selection and emergence of other RV species, such as RVB and RVC^23,24^. The high frequency of RVB found in this study contributes to this hypothesis since the sows of all pig herds described in this study were routinely vaccinated against enteric pathogens, including RVA. Molinari et al.^6^ reported similar findings describing the detection of multiple RV groups in single and mixed infections in an unusual post-weaning porcine diarrhoea outbreak in a pig herd vaccinated with the RVA OSU strain in Brazil.

It is known that RVA species are shed in large quantities in stools of infected animals during episodes of diarrhoea, with viral loads of approximately 10^12^ viral particles per gram of faeces^25^. In this study, the RVB viral load found was up to 8.5 × 10^8^ genomic copies/g of faeces, suggesting that lower quantities of RVB are shed during the acute period of infection compared to RVA. However, few virions are needed to cause disease in susceptible animals^4^ and the transmission of RV infection occurs predominantly by the faecal-oral route, suggesting that environmental contamination contributed to the various outbreaks that occurred during the total outbreak period described in the present study.

The low prevalence of RVB reported worldwide may not be a consequence of a low infection rate but rather due to diagnostic gaps^26^. Some techniques, such as ssPAGE and EM, have low sensitivity, presenting an LOD of approximately 10^6^ particles/mL^27^. In this study, the prevalence of RVB found by conventional RT-PCR was higher than by ssPAGE. Moreover, the viral loads found in this study varied from 7.6 × 10^2^ to 8.5 × 10^8^ genomic copies/g of faeces, and most of the positive samples on ssPAGE presented viral loads above 10^6^ genomic copies/g of faeces (data not shown). Therefore, the use of more sensitive diagnostic techniques is necessary to avoid false-negative results. Molecular techniques, such as RT-PCR and qRT-PCR, usually present high sensitivity and are suitable assays to detect RVB from porcine biological samples^6,7,23^.

Unlike RVA, RVB does not adapt and propagate in cell culture, which has hindered its serological and molecular characterization, as well as the development of vaccines against RVB disease. In 1996, the isolation of a putative RVB strain in cultured cells, designated the SKA-1 strain, was reported^28^. However, some years later, the same research group found that this strain was actually more closely related to RVH^29^. In this study, our attempts to isolate RVB in cell culture were unsuccessful. Nevertheless, by EM, we identified RV-like particles in the same sample that was submitted for virus isolation in cell culture. Electron microscopy lacks specificity because it cannot differentiate among RV species^2^; however, the submitted sample tested negative in the RT-PCR for all RV species and other viral agents, except for RVB, suggesting that the virus identified was probably RVB. In addition, the VP7 protein of this RVB strain was amplified, and we could determine its G genotype (G20).

Until recently, information regarding the genome of porcine RVB strains was restricted to only a few proteins^11,16,30–33^. The classification for G genotypes was first proposed by Kuga et al.^16^, who analysed the VP7 gene of Japanese RVB strains. Using nt cutoff values of 67 and 76%, the authors established five G genotypes, divided into 12 clusters^16^. After that, based on the analysis of RVB strains from the USA, Marthaler et al.^11^ proposed a modification of the nt cutoff value to 80%, establishing twenty G genotypes. Based on this new cutoff value, Lahon et al.^14^ and Molinari et al.^34^ each described new RVB G genotypes, G21 and G22, respectively. More recently, Shepherd et al.^12^ described the whole genome of USA RVB strains, suggesting and updating cutoff values for all RVB gene segments, including those that had not been established yet. According to this most recent classification, 26 G genotypes for RVB were defined. In addition, the previously reported Brazilian G22 strain^34^ was reclassified into the G24 genotype.

Only two VP7 RVB strains have been described in Brazil thus far, BR31 (G14) and BR62 (G24)^6,34^. In this study, we report 16 new Brazilian VP7 RVB wild-type strains. The phylogenetic analysis performed with the complete VP7 gene of the RVB strains identified in this study revealed that four different G genotypes were circulating in the same Brazilian geographic region (Central-West). The G16 genotype was the most prevalent, followed by the G12, G20, and G14 genotypes. Marthaler et al.^11^ also reported G16 as the most prevalent G genotype in RVB field strains in the USA.

Interestingly, in the two pig herds from which we performed longitudinal phylogenetic analysis, two different G genotypes were detected over the sampling period. In pig herd C, the G16 genotype was detected within the first to third samplings, while in the fourth sampling, which occurred 8 months later, the G12 genotype was found. Similarly, in pig herd D, the genotype found in the first sampling was G14, and the G16 genotype was found circulating in the same herd ten months later. These findings suggest a high variation in G genotypes circulating in Brazil.

RVB appears to be more diverse than RVA and RVC when considering the number of genotypes in swine hosts. According to Shepherd et al.^12^, 17 G genotypes of RVB have been identified in pigs compared with 12 and 15 G genotypes of RVA and RVC, respectively. Some authors even suggested that swine species might be the original natural host of RVB, strongly contributing to the genetic diversity of this RV species^16,35^.

Since RVB has been identified as an important enteric pathogen of pigs, it is essential to incorporate screening for RVB along with RVA and RVC in the diagnostic routine of swine enteric diseases. More information about the epidemiology of porcine RVB is needed to better understand its pathogenicity and prevent RVB infections, since to date there are no vaccines available for this RV species.

## Conclusion

This study reports RVB as the primary causative agent of neonatal diarrhoea outbreaks in newborn piglets. RVB was associated with marked villous atrophy and blunting in the jejunum of affected piglets, inducing malabsorptive diarrhoea and leading to high mortality rates. We suggest that the reported repeated outbreaks in these pig herds were associated with environmental contamination since we found mild to high genomic copies of RVB in piglet faeces. Additionally, the rates of RVB diagnosis in diarrhoea outbreaks reported in this study were much higher than those described in previous studies, mainly in single infections. Moreover, the variety of G genotypes identified in these outbreaks suggests that genetic diversity among porcine RVB field strains may be underestimated. Further studies are necessary to investigate the prevalence of RVB in other regions of Brazil and in other countries, as well as to identify the genotypes circulating worldwide.

## Materials and methods

### Herds and samples

Neonatal diarrhoea outbreaks occurred from July 2017 to August 2018 in piglet production units from eight pig herds located in a single state of the Central-West region of Brazil. The all-in-all-out production system was used for all the pig herds, and good nutritional and health management practices were used, including the addition of vitamin and probiotic supplements and heat placement for newborn piglets. All sows were vaccinated with a commercial vaccine containing the RVA OSU (G5P [7]) strain, *Escherichia coli* (K88, K99, F41 and 987P) and *Clostridium perfringens* types C and D, following the manufacturer’s recommendations. Nevertheless, the outbreaks were unresponsive to wide-spectrum antibiotic therapy, exhibiting high mortality (10 to 50%) and morbidity (35 to 50%) rates. Piglets up to 20 days of age, mostly newborn piglets, from all herds were affected and exhibited dehydration and watery diarrhoea, with the onset of symptoms usually at one day after birth.

For four of the eight pig herds affected by the diarrhoea outbreak, four or five sampling were collected at different times, with 1–8 months between each sampling, enabling us to perform a longitudinal analysis of these herds.

A total of 90 biological samples, including 77 diarrheic faeces and 13 fragments of the small intestines from suckling piglets (1 to 20 days of age), were subjected to analysis for the identification of enteric viruses. All intestinal fragments were immersed in 10% buffered formalin solution and then routinely processed for histopathologic evaluation with haematoxylin and eosin (H&E) staining.

### Nucleic acid extraction, PAGE, and conventional RT-PCR

The nucleic acid was extracted from 10% faecal/intestine suspensions in phosphate-buffered saline (PBS), pH 7.2, using a combination of phenol/chloroform/isoamyl alcohol (25:24:1) and silica/guanidine isothiocyanate methods, described by Alfieri et al.^36^. The nucleic acid was eluted in 50 µL of UltraPure DEPC-treated water (Invitrogen Life Technologies, Carlsbad, CA, USA) and stored at − 80 °C until further use. Aliquots of ultrapure sterile water were included in all nucleic acid extraction and the following procedures as negative controls.

All the samples were tested by PAGE assay to verify the presence of RV ^13^. The extracted nucleic acid was also subjected to conventional RT-PCR assay to investigate and confirm the presence of RV species A, B, C, and H, as well as other porcine enteric viruses, such as SDCoV, PEDV, TGEV, SVV, and PEV. The gene targets and the RT-PCR products of each virus are described in Table 2. The amplified products were analysed by 2% agarose gel electrophoresis in TBE buffer; the gels were stained with 0.5 µg/mL of ethidium bromide and visualized under ultraviolet light.

**Table 2:** Gene targets and respective RT-PCR products used to identify enteric viruses in pig samples.

Virus	Viral gene	Primer sequence (5′–3′)	Primer position	RT-PCR product (bp)	References
RVA	VP4 VP7	Fw-TGGCTTCGCCATTTLATAGACA Rv-ATTTCGGACCATTTATAACC Fw-GGCTTTAAAAGAGAGAATTTCCGTCTGG Rv-GGTCACATCATACAATTCTAATCTAAG	12–33 868–887 1–21 1038–1062	876 1062	^39^ ^40^
RVB	NSP2	Fw-CTATTCAGTGTGTCG TGAGAGG Rv-CGAAGCGGGCTAGCTTGTCTGC	1–18 434–451	434	^41^
RVB	VP7	Fw-GCGTTGCCACTGCTTCTC Rv-TTTTTATTGGCTTCGGCTACTC	18–35 796–817	800	^15^
RVC	VP6	Fw-GGCTTTAAAAATCTCATTCA Rv-CCTCTAGTTGATTGAACATA	1–20 251–270	270	^42^
RVH	VP6	Fw-ACCAGGTGGAGCAACAAACA Rv-CAGTGCGTGACCAGATCTCA	529–549 1225–1244	716	^43^
SDCoV	M	Fw-ATCCTCCAAGGAGGCTATGC Rv-GCGAATTCTGGATCGTTGTT	67–87 540–560	493	^44^
TGEV	S	Fw-GTGGTTTTGGTYRTAAATGC Rv-CACTAACCAACGTGGARCTA	16–35 855–874	859	^45^
PEDV	S	Fw-TTCTGAGTCACGAACAGCCA Rv-CATATGCAGCCTGCTCTGAA	1466–1485 2097–2116	651	^45^
SVV	VP1	Fw-ACTGACACCGATTTCTCTG Rv-CTAAAGTAAGTGAAACAGGC	2730–2749 3026–3046	316	^46^
PEV	5′ NTR	Fw-CAAGCACTTCTGTTTCCCCGG Rv-GTTAGGATTAGCCGCATTCAGGGG	197–271 486–509	313	^47^

### Transmission electron microscopy

Two faecal samples positive for RVB in the RT-PCR assay were subjected to EM examination. The 10% (w/v) faecal suspensions in PBS solution were clarified by centrifugation at 1500 × *g* for 30 min. Then, a 5 µL drop of each sample was applied onto a 300-mesh formvar-coated cooper grid (Electron Microscopy Sciences, Hatfield, PA, USA) and negatively stained with 3% phosphotungstic acid for 90 s. Grids were prepared in duplicate and examined at 80 kV with a Tecnai-G2 transmission electron microscope (FEI Company, Eindhoven, The Netherlands).

### Virus isolation

Attempts at virus isolation from two RVB-positive faecal samples in the RT-PCR assay were performed in a monolayer of MA-104 cells, which were grown in Dulbecco's Modified Eagle's medium (DMEM; Gibco BRL, Grand Island, NY, USA) supplemented with 10% foetal calf serum (Gibco BRL, Carlsbad, CA, USA).

The inoculum was prepared as follows. A 10% (w/v) faecal suspension in PBS solution was pretreated with 3 × penicillin, streptomycin, and fungizone (Gibco Antibiotic‐Antimycotic, Invitrogen Life Technologies, Carlsbad, CA, USA) for 24 h. Then, the suspension was supplemented with 10 µg/mL trypsin and incubated at 37 °C for 30 min. Before inoculation, the confluent monolayer cells were washed 2–3 times with serum-free DMEM. Then, 1 mL of the treated suspension was inoculated onto the cell monolayer. The inoculum was allowed to adsorb at 37 °C for 3 h in a temperature‐controlled rocker platform. The inoculated MA-104 cells were maintained with serum-free DMEM supplemented with 0.5 µg/mL trypsin in a CO_2_ incubator (Thermo Electron Corporation, Marietta, OH, USA) and inspected daily for CPE.

### qRT-PCR assay

All RVB-positive samples in the RT-PCR assay from these outbreaks were subjected to qRT-PCR assay to quantify the corresponding viral load. The qRT-PCR assay was developed in this study to quantify RVB RNA in biological samples.

The primers qRVB1174-Fw (5′-TGTTAGTATCCGCATTTGCTG-3′) and qRVB1269-Rv (5′-GGGTTTTATTGCTTATTTTTTCG-3′) were designed to amplify a 95 bp fragment of a specific region of the porcine RVB VP6 gene. The probe was defined as qRVB1206-pb (5′-FAM-TCCGGCGTCAGCTCCCAAAGG-MGB-3′) (Applied Biosystems, Foster City, CA, USA).

The qRT-PCR assay was performed in a 7500 Fast Real-Time PCR System (Applied Biosystems, Foster City, CA, USA) using the SuperScript III Platinum One-Step Quantitative RT-PCR System (Invitrogen Life Technologies, Carlsbad, CA, USA). The qRT-PCR master mix was prepared following the manufacturer’s instructions with a final volume of 25 µL, including 50 nM Rox Reference Dye, 400 nM each forward and reverse primer, 200 nM probe and 5 µL of genomic template. All the samples were tested in triplicate. The cycling conditions included reverse transcription for 15 min at 50 °C, *Taq* activation for 2 min at 95 °C, followed by 40 cycles of 15 s at 95 °C, and 30 s at 60 °C. Sterile ultrapure water was used as a negative control in all reactions.

The number of genome copies present in the RVB-positive samples was estimated by comparing the sample cycle threshold (*C*_t_) value to standard curves. To generate the standard curves, a 95 bp-RVB VP6 fragment was cloned into the pCR 4-TOPO vector (Invitrogen Life Technologies, Carlsbad, CA, USA), according to the manufacturer’s instructions, and ligation products were transformed into TOP10 competent *E. coli*. After transformation, *E. coli* insert-containing cultures were extracted and purified by PureLink Quick Plasmid Miniprep Kit (Invitrogen Life Technologies, Carlsbad, CA, USA) and then quantified by Qubit Fluorometer (Invitrogen Life Technologies, Eugene, OR, USA). Tenfold serial dilutions of the RVB VP6 clone containing 1.34 × 10^7^ to 1.34 × 10^0^ copies/µL were used to generate the standard curve and to estimate the LOD of the qRT-PCR assay. The final concentration in the samples was adjusted based on the volume of nucleic acids used and was expressed per gram of faeces/intestine tissue.

### Sequencing and phylogenetic analysis

To identify the G genotypes of the porcine RVB strains, a VP7-specific RT-PCR was carried out as described by Kuga et al.^16^. Sixteen RVB-positive faecal samples were selected for sequence analysis, and at least one RVB field strain was chosen from each pig herd. The RT-PCR products of approximately 800 bp were purified using the PureLink Quick Gel Extraction and PCR Purification Combo Kit (Invitrogen Life Technologies, Carlsbad, CA, USA), quantified with a Qubit Fluorometer (Invitrogen Life Technologies, Eugene, OR, USA), and sequenced using an ABI 3500 Genetic Analyser with a BigDye Terminator v3.1 Cycle Sequencing kit (Applied Biosystems, Foster City, CA, USA).

Sequence quality analyses and consensus were conducted using Phred and CAP3 software (https://aspargin.cenargen.embrapa.br/phph/). Sequence similarity searches were performed with sequences deposited in GenBank using the Nucleotide Basic Local Alignment Search Tool (BLASTn) (https://blast.ncbi.nlm.nih.gov/Blast.cgi). A phylogenetic tree based on nucleotide sequences of the VP7 gene of porcine RVB strains described in this study and representative strains of the 26 previously identified RVB G genotypes^12^ was created using the neighbour-joining method based on the Kimura two-parameter model, which provided statistical support by bootstrapping with 1,000 replicates in MEGA 7 software^37^. BioEdit software, version 7.2.5, was used to construct the sequence identity matrix^38^. The nt cutoff value used to classify the RVB strains into G genotypes was 80%, as suggested by Shepherd et al.^12^ (accession numbers of all RVB G genotype strains used to construct the phylogenetic tree and the identity matrix can be found as Supplementary Table S3 online).

### Nucleotide sequences accession numbers

The nucleotide sequences described in this study were deposited in the GenBank database under the following accession numbers: MN540126 to MN540141.

### Ethical approval

This study was submitted to the Ethics Committee on Animal Experiments of the Universidade Estadual de Londrina and approved under the identification number 11363.2015.16. All applicable international, national, and/or institutional guidelines for the care and use of animals were followed.

## Supplementary Information

Supplementary Information.
