# Supplementary material for: Porcine rotavirus B as primary causative agent of diarrhea outbreaks in newborn piglets
Source: Sci Rep. 2020 Dec 15;10:22002. doi: 10.1038/s41598-020-78797-y (PMC7738533; doi:10.1038/s41598-020-78797-y)
Supplement: Supplementary file 1 — Supplementary Information. [file 41598_2020_78797_MOESM1_ESM.pdf]

Porcine Rotavirus B as Primary Causative Agent of Diarrhea Outbreaks in Newborn Piglets

Authors: Flavia Megumi Miyabe, Alais Maria Dall Agnol, Raquel Arruda Leme, Thalita Evani Silva Oliveira, Selwyn Arlington Headley, Thiago Fernandes, Admilton Gonçalves de Oliveira, Alice Fernandes Alfieri, Amauri Alcindo Alfieri.

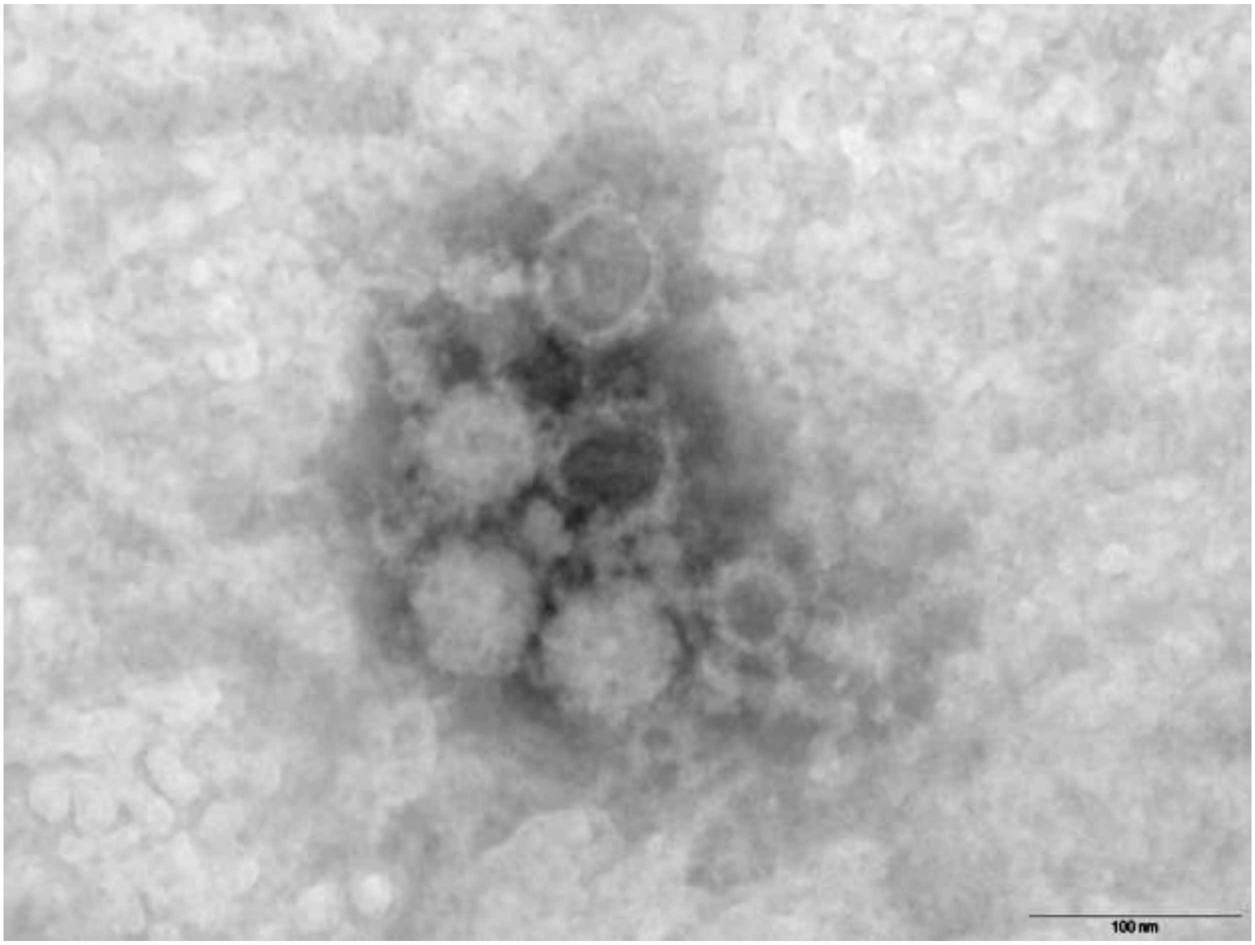

Supplementary Figure S1. Negative staining of Rotavirus-like particles in a faecal sample analysed by transmission electron microscopy. Bar, 100 nm. Magnification, ×100,000.

Supplementary Table S2. Identity matrix of G genotypes of RVB strains described in this study and representative strains of the 26 previously identified RVB G genotypes.

	GO-949	GO-951	GO-1099	GO-1113	GO-936	GO-908	GO-923	GO-968	GO-970	GO-983	GO-1045	GO-1053	GO-1119	GO-1123	GO-1127	GO-992
**G1**	58	58	57	58.3	55.7	58.8	59.2	59.2	59.2	59	58.8	58.8	59	58.6	58.6	54.7
**G2**	61.7-62.4	61.8-62.3	61.8-62.6	61.4-62.7	60.1-60.7	61-61.7	59.7-60.5	59.7-60.5	59.7-60.5	59.5-60.4	61-61.8	60.8-61.7	59.8-60.7	60.8-61.8	60.8-61.8	57.5-57.9
**G3**	60.2-61.8	60.2-61.8	59.2-60.4	59.7-60.7	57.9-58.9	57.1-58.4	57.2-57.9	57.2-57.9	57.2-57.9	57.3-58.1	56.9-58.5	57.1-58.4	57.3-58.1	57.5-58.5	57.5-58.7	56.5-57.3
**G4**	65.3	65.2	66	64	66.8	60.4	60.7	60.7	60.7	60.8	60.8	60.7	60.7	60.8	60.8	60.7
**G5**	61.8-62	61.7-61.8	60.5-61	60.5-61	59.8-60.1	59.5-60	59.5-59.7	59.5-59.7	59.5-59.7	59.7-59.8	59.7-60.1	59.8-60.2	59.7-59.8	59.7-60.1	59.7-60.1	56.9-57.2
**G6**	62-62.7	62-62.6	60.5-61.3	61.3-62.1	60.5-60.8	60.5-61.1	60.1-60.4	60.1-60.4	60.1-60.4	60.2-60.5	60.8-61	60.7-61.1	60.2-60.5	61-61.5	61-61.5	57.1-57.5
**G7**	62.7	62.7	62.7	62.1-62.3	62.4-63.4	60.8-61.4	61-62	61-62	61-62	61.1-61.2	61.3	61.1-61.4	61-62	60.8-61.1	60.8-61.1	57.5-58.9
**G8**	61.8-62.8	61.7-62.7	62.1-64.7	61.7-62.7	60.2-62.3	59.7-61.1	60.4-62.3	60.4-62.3	60.4-62.3	60.5-62.1	59.4-61.7	59.5-61.8	60.2-62.1	60.1-62.4	60.1-62.4	59.5-61
**G9**	62.1	62.1	62	60.8	62	59.7	60.1	60.1	60.1	60	59.4	59.5	60.1	59.8	59.8	58.1
**G10**	61	60.8	60.8	61.5	61.7	62	61.4	61.4	61.4	61.5	62.4	62.3	61.5	62.4	62.4	59.5
**G11**	68.2-71.1	68.1-71	69.5-71.1	69.4-71.6	72.4-73.2	70.5-72.7	70.8-72.9	70.8-72.9	70.8-72.9	70.7-72.7	70.5-72.7	70.7-72.9	70.8-72.9	71-73	71-73	66.9-71
**G12**	77.5-80.4	77.4-80.3	77.2-80.3	77.1-80.3	72.9-74	71.3-73.9	71-73.7	71-73.7	71-73.7	70.8-73.6	71.6-73.6	71.4-74	71.1-73.9	71.4-74.2	71.4-74.2	68.1-71.1
**G13**	73	72.9	72.7	74.3	74.6	72.3	72.7	72.7	72.7	72.6	72.3	72.4	72.9	72.3	72.3	71.7
**G14**	72.3-74.9	72.4-74.8	72.9-75.2	72.9-74.8	80-84.4	73.6-74.8	73.7-74.5	73.7-74.5	73.7-74.5	73.6-74.6	73.5-75.2	73.6-75.1	73.7-74.9	73.9-75.4	73.9-75.4	69.7-71.7
**G15**	71.9	71.7	72.6	73.9	77	74	74.2	74.2	74.2	74	73.9	74	74.3	74.3	74.3	70.1
**G16**	71.4-72.9	71.3-72.7	70.8-73.3	71.9-73.2	74.3-75.6	82-83.4	81.5-82.5	81.5-82.5	81.5-82.5	81.3-82.3	82.3-83.6	82.2-83.5	81.6-82.6	82.3-83.9	82.3-83.9	71.6-73.6
**G17**	70.4-71.3	70.3-71.1	72.4-72.9	71.1-72.3	74.5	75.4	75.4-75.6	75.4-75.6	75.4-75.6	75.2-75.5	75.4-75.6	75.5	75.5-75.8	75.6-76.1	75.6-76.1	70.8-71.4
**G18**	70.1-72.6	70-72.4	69.4-72.1	70.8-71.6	72.7-73.9	73-75.4	73-74.6	73-74.6	73-74.6	72.9-74.5	73.2-75.5	73.3-75.4	73.2-74.8	72.3-75.8	72.3-75.8	72.1-72.4
**G19**	59.3	59.3	58.8	60.2	59.5	60.4	60.4	60.4	60.4	60.5	60.6	60.5	60.5	60.8	60.8	62
**G20**	69.2-71	69.1-70.1	67.5-71.1	68.8	68.4-70.5	67.8-71.6	67.5-71.4	67.5-71.4	67.5-71.4	67.6-71.6	67.8-71.7	67.9-71.6	67.5-71.4	67.9-71.7	67.9-71.7	81.3-85.4
**G21**	63.9	63.9	64.4	62.7	64.2	63.3	62	62	62	61.8	63.4	63.3	62.1	63.3	63.3	60.5
**G22**	69.2	69.2	68.8	67.6	68.9	69.8	70.3	70.3	70.3	70.1	70	69.8	70.4	69.8	69.8	72.9
**G23**	62.4	62.3	62.7	62.3	61.8	63	61.8	61.8	61.8	62	63	63	62	62.8	62.8	59.7
**G24**	67.2	67.1	67.2	67.2	66.6	63.7	63.7	63.7	63.7	63.6	64.2	64	63.7	63.9	63.9	63.3
**G25**	73.2	73	73.7	72.1	72.6	73.5	72.7	72.7	72.7	72.6	73.3	73.5	72.9	73.7	73.7	69.1
**G26**	66.6	66.6	68.1	66.6	66.8	68.2	68.6	68.6	68.6	68.8	68.2	68.3	68.8	68.8	68.8	70.1

Supplementary Table S3. Accession number of the G genotypes of RVB strains used to construct the phylogenetic tree and the identity matrix. The Brazilian porcine RVB strains described in the present study are marked with filled circles.

RVB G genotype	Accession number	Strain
G1	D00911	RVB/Rat-hhp/USA/IDIR/1984/G1P[X]
G2	AY539856	RVB/Human-wt/CHN/WH-1/2002/G2P[X]
	AF184083	RVB/Human-wt/IND/CAL-1/1998/G2P[X]
G3	AB016818	RVB/Cow-wt/JPN/Nemuro/1997/G3P[X]
	KY689695	RVB/Goat-wt/USA/Minnesota-1/2016/G3P[3]
G4	AB490451	RVB/Pig-wt/PB-70-H5/2007/G4P[X]
G5	AF531910	RVB/Cow-wt/IND/DB176/2001/G5P[3]
	AF529214	RVB/Cow-wt/IND/DB180/2001/G5P[X]
G6	AB490417	RVB/Pig-wt/JPN/PB-F18/2001/G6P[X]
	JQ043782	RVB/Pig-wt/USA/MN09-35/2009/G6P[X]
G7	AB490423	RVB/Pig-wt/JPN/PB-S15/2002/G7P[X]
	AB490454	RVB/Pig-wt/JPN/PB-72-I2/2007/G7P[X]
G8	AB490436	RVB/Pig-wt/JPN/PB-S49-13/2003/G8P[X]
	JQ043773	RVB/Pig-wt/USA/NE09-26/2009/G8P[X]
G9	AB490448	RVB/Pig-wt/JPN/PB-68-E4/2007/G9P[X]
G10	JQ043781	RVB/Pig-wt/USA/MO09-34/2009/G10P[X]
G11	JQ043807	RVB/Pig-wt/USA/OH09-60/2009/G11P[X]
	JQ043767	RVB/Pig-wt/USA/MN09-20/2009/G11P[X]
G12	AB490426	RVB/Pig-wt/JPN/PB-S24-11/2002/G12P[X]
	AB490429	RVB/Pig-wt/JPN/PB-S40-1/2003/G12P[X]
	JQ043809	RVB/Pig-wt/USA/MN09-62/2009/G12P[X]
	JQ043768	RVB/Pig-wt/USA/MO09-21/2009/G12P[X]
	JQ043776	RVB/Pig-wt/USA/NC09-29/2009/G12P[X]
	MN540129	RVB/Pig-wt/BRA/GO-949/2017/G12P[X]●
	MN540130	RVB/Pig-wt/BRA/GO-951/2017/G12P[X]●
	MN540137	RVB/Pig-wt/BRA/GO-1099/2018/G12P[X]●
	MN540138	RVB/Pig-wt/BRA/GO-1113/2018/G12P[X]●
G13	AB490445	RVB/Pig-wt/JPN/PB-23-44/2005/G13P[X]
G14	KX376976	RVB/Pig-wt/BRA/BR31/2012/G14P[X]
	AB490425	RVB/Pig-wt/JPN/PB-S22-3/2002/G14P[X]
	JQ043806	RVB/Pig-wt/USA/MN09-59/2009/G14P[X]
	JQ043756	RVB/Pig-wt/USA/AR09-9/2009/G14P[X]
	MN540128	RVB/Pig-wt/BRA/GO-936/2017/G14P[X]●
G15	AB490435	RVB/Pig-wt/JPN/PB-S49-2/2003/G15P[X]
G16	AB490446	RVB/Pig-wt/JPN/PB-68-C17/2007/G16P[X]
	JQ043772	RVB/Pig-wt/USA/IL09-25/2009/G16P[X]
	JQ043786	RVB/Pig-wt/USA/OK09-39/2009/G16P[X]

G16	JQ043814	RVB/Pig-wt/USA/IA09-67/2009/G16P[X]
	MG272202	RVB/Pig-wt/USA/IL20/2013/G16P[5]
	MG272190	RVB/Pig-wt/USA/IL8/2013/G16P[4]
	MG272204	RVB/Pig-wt/USA/KS1/2012/G16P[4]
	MN540126	RVB/Pig-wt/BRA/GO-908/2017/G16P[X]●
	MN540127	RVB/Pig-wt/BRA/GO-923/2017/G16P[X]●
	MN540131	RVB/Pig-wt/BRA/GO-968/2017/G16P[X]●
	MN540132	RVB/Pig-wt/BRA/GO-970/2017/G16P[X]●
	MN540133	RVB/Pig-wt/BRA/GO-983/2017/G16P[X]●
	MN540135	RVB/Pig-wt/BRA/GO-1045/2018/G16P[X]●
	MN540136	RVB/Pig-wt/BRA/GO-1053/2018/G16P[X]●
	MN540139	RVB/Pig-wt/BRA/GO-1119/2018/G16P[X]●
	MN540140	RVB/Pig-wt/BRA/GO-1123/2018/G16P[X]●
	MN540141	RVB/Pig-wt/BRA/GO-1127/2018/G16P[X]●
G17	JQ043799	RVB/Pig-wt/USA/OK09-51/2009/G17P[X]
	MF522294	RVB/Pig-wt/USA/MS-32/2012/G17P[X]
G18	AB490443	RVB/Pig-wt/JPN/PB-23-5/2005/G18P[X]
	JQ043810	RVB/Pig-wt/USA/MN09-63/2009/G18P[X]
G19	KJ613649	RVB/Pig-wt/IND/AN142129/2013/G19P[X]
G20	AB490418	RVB/Pig-wt/JPN/PB-Taiheiyo/2000/G20P[X]
	AB490427	RVB/Pig-wt/JPN/PB-S26-1/2002/G20P[X]
	KX362394	RVB/Pig-wt/VNM/14176_8/2012/G20P[X]
	JQ043760	RVB/Pig-wt/USA/MO09-13/2009/G20P[X]
	JQ043763	RVB/Pig-wt/USA/IL09-16/2009/G20P[X]
	MN540134	RVB/Pig-wt/BRA/GO-992/2017/G20P[X]●
G21	KJ613650	RVB/Pig-wt/IND/AN142530/2013/G21P[X]
G22	MF072691	RVB/Pig-wt/BRA/BR62/2018/G22P[X]
	MF522360	RVB/Pig-wt/USA/MN-98/2014/G22P[X]
G23	MF522387	RVB/Pig-wt/USA/MN-125/2014/G23P[X]
	MF522388	RVB/Pig-wt/USA/MN-126/2014/G23P[X]
G24	MF522389	RVB/Pig-wt/USA/MN-127/2014/G24P[X]
	KX362405	RVB/Pig-wt/VNM/14177_18/2012/G24P[X]
G25	MF522325	RVB/Pig-wt/USA/OK-63/2013/G25P[X]
G26	AB490449	RVB/Pig-wt/JPN/PB-68-G4/2009/G26P[X]
